# Doxycycline Regulated Induction of AKT in Murine Prostate Drives Proliferation Independently of p27 Cyclin Dependent Kinase Inhibitor Downregulation

**DOI:** 10.1371/journal.pone.0041330

**Published:** 2012-07-23

**Authors:** Hongyun Wang, Youyuan Xu, Zi Fang, Sen Chen, Steven P. Balk, Xin Yuan

**Affiliations:** Hematology-Oncology Division, Department of Medicine, Beth Israel Deaconess Medical Center, Harvard Medical School, Boston, Massachusetts, United States of America; Technische Universität München, Germany

## Abstract

The PI3 kinase/AKT pathway has been shown to increase degradation of the p27 cyclin dependent kinase inhibitor through phosphorylation of consensus AKT sites on p27 and SKP2, and AKT driven proliferation may be checked by feedback mechanisms that increase p27 expression and induce senescence. However, these AKT sites are not conserved in mouse, and it has not been clear whether AKT negatively regulates murine p27. Transgenic mice with a probasin promoter controlled prostate specific reverse tetracycline transactivator (ARR2Pb-rtTA) were generated and used to achieve doxycycline inducible expression of a tetracycline operon regulated constitutively active myristoylated AKT1 transgene (tetO-myrAKT). Doxycycline induction of myrAKT occurred within 1 day and rapidly induced proliferation (within 4 days) and the development of prostatic intraepithelial neoplasia (PIN) lesions in ventral prostate, which did not progress to prostate cancer. Cells in these lesions expressed high levels of p27, had increased proliferation, and there was apoptosis of centrally located cells. Doxycycline withdrawal resulted in apoptosis of cells throughout the lesions and rapid clearing of hyperplastic glands, confirming *in vivo* the critical antiapoptotic functions of AKT. Significantly, analyses of prostates immediately after initiating doxycycline treatment further showed that p27 expression was rapidly increased, coincident with the induction of myrAKT and prior to the development of hyperplasia and PIN. These findings establish *in vivo* that murine p27 is not negatively regulated by AKT and indicate that proliferation in PI3 kinase/AKT pathway driven mouse models is mediated by p27 independent mechanisms that may be distinct from those in human. Further studies using prostate specific doxycycline regulated transgene expression may be useful to assess the acute effects of inducing additional transgenes in adult murine prostate epithelium, and to assess the requirements for continued transgene expression in transgene induced tumors.

## Introduction

PTEN expression is very frequently downregulated through deletion, mutation or other mechanisms in prostate cancer (PCa), and *Pten* loss is common in higher grade primary and advanced metastatic PCa. Mice with prostate epithelium specific *Pten* deletion develop intraepithelial hyperplasia and dysplasia (prostatic intraepithelial neoplasia, PIN), but there is generally a long latency period before these lesions progress to invasive cancer. Recent studies indicate that this latency is due to induction of a p53-dependent senescence pathway, with *Pten* loss on a p53 deficient background causing a marked acceleration in PCa development [1], [2], [3], [4].

PTEN loss enhances PI3 kinase signaling and activates its major downstream effector, AKT. Similar to the effects of *Pten* loss, mice with prostate epithelium specific expression of a constitutively active myristoylated AKT transgene (myrAKT) develop PIN, although these myrAKT mediated lesions do not progress to invasive cancer [5]. This may reflect some functional differences between myrAKT and endogenous AKT that is activated physiologically downstream of *Pten* loss, or may reflect additional AKT independent mechanisms by which *Pten* loss is driving tumor progression. In either case, as observed with *Pten* loss, myrAKT mediated PIN lesions undergo cellular senescence that is correlated with high level expression of the cyclin dependent kinase inhibitor p27 [6]. Significantly, decreased p27 correlates with more aggressive behavior in human PCa [7], and the development of PCa in mouse prostate with *Pten* loss is markedly accelerated on p27 deficient backgrounds [8]. Similarly, p27 deficient mice expressing myrAKT in prostate epithelium develop invasive PCa [6], indicating that both p27 and p53 are functioning to check the progression of PIN to invasive cancer, as had been reported previously in RB deficient tumor models [9], [10].

The Cre mediated loss of *Pten* and the induction of myrAKT in these mouse PCa models are controlled by elements from the rat probasin promoter, which is regulated by androgen and activated specifically in prostate luminal epithelium [11]. To study the consequences of acute and chronic oncogene activation and silencing in adult prostate, this report describes generation of transgenic mice expressing a reverse tetracycline transactivator (rtTA) [12] under the control of elements from the rat probasin promoter (ARR2Pb) [11], and their use to control expression of a tetracycline operon regulated myristoylated AKT1 transgene (tetO-myrAKT) [13].

## Results

### Doxycycline Mediated Induction of Activated AKT and PIN in Murine Prostate

**Figure 1:**
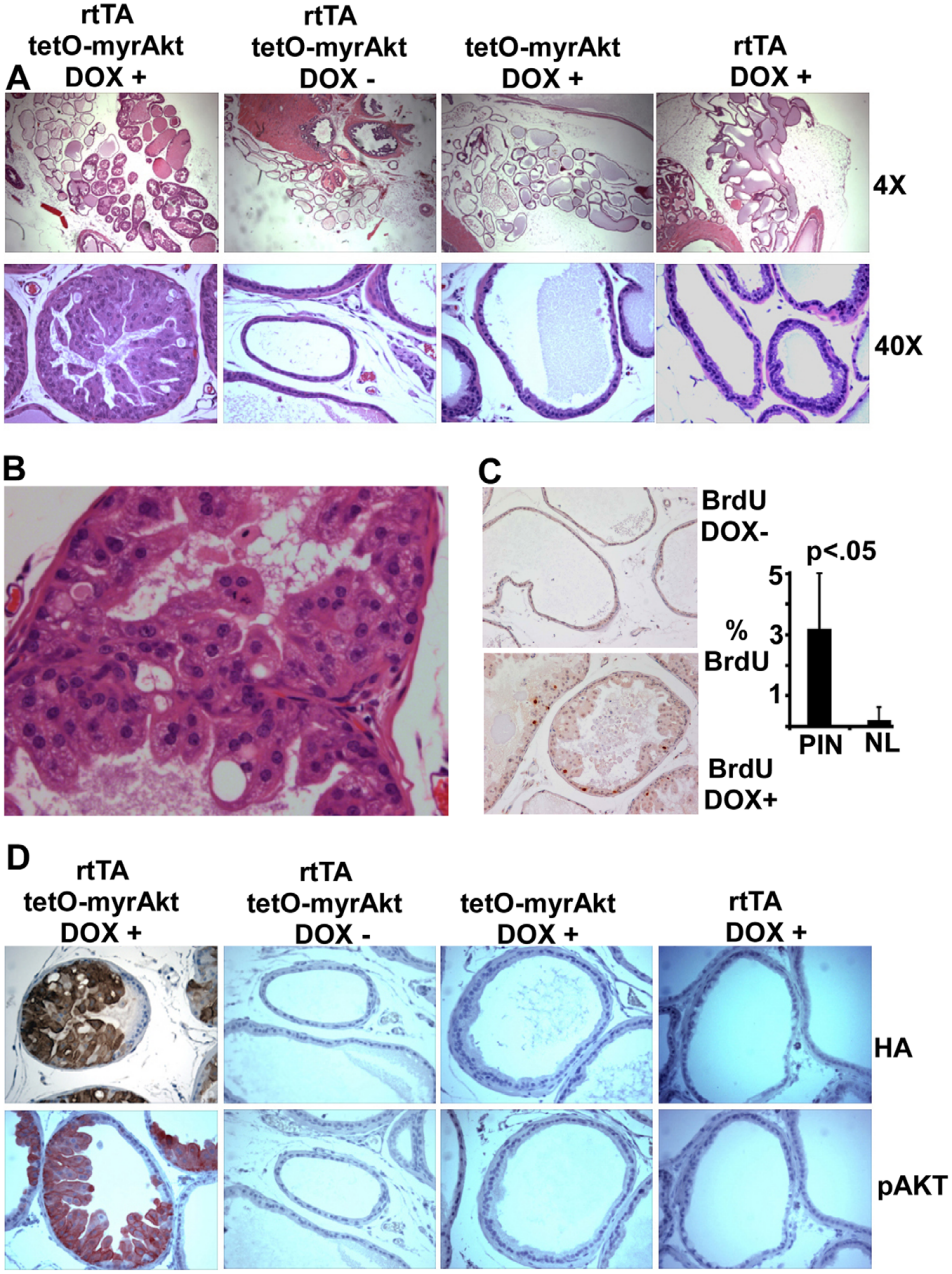
Doxycycline induces myrAKT expression and development of PIN. (**A**) Adult double or single transgenic mice were treated as indicated for 8 weeks and prostates were analyzed for histology. Ventral prostates are shown; no clear alterations were found in dorsal, lateral, or anterior prostate. (**B**) Higher power view of PIN lesion is shown. (**C**) Double transgenic mice induced with doxycycline for 8 weeks were injected intraperitoneally with BrdU (0.1 ml of 10 mg/ml solution in sterile PBS) at 4 hours prior to sacrifice, and S-phase cells were then assessed by anti-BrdU immunohistochemistry. Graph show average % of BrdU positive cells counted in 10 high power views (600×) in PIN lesions or in normal glands (NL) from uninduced control mice. (**D**) Sequential sections were immunostained for total HA-tagged AKT and phospho-Akt.

Sixteen founder lines transmitting the rtTA transgene were crossed with a tetO-β-galactosidase reporter strain and prostates from adult (∼8 week) double and control single transgenic mice treated with doxycycline were examined. Histochemical staining detected weak β-galactosidase enzyme activity in the ventral prostate of several lines, with line 42 yielding the strongest and most consistent staining (data not shown). To determine whether the rtTA in this line could drive functionally significant levels of a tetO regulated oncogene, we bred this line with mice containing a tetO-myrAKT transgene (HA-epitope tagged myrAKT1) [13]. Histological examination of double transgenic mice after 8 weeks on doxycycline revealed hyperplasia and dysplasia in ventral prostate (Fig. 1A), with affected glandular acini showing multiple disorganized layers and cribiforming, intraepithelial lumens, disrupted cellular polarity, nuclear atypia, apoptotic bodies and fragment accumulation (Fig. 1B). Anti-BrdU immunostaining of prostates from mice injected intraperitoneally with BrdU at 4 hours prior to sacrifice confirmed a marked increase in proliferation (Fig. 1C). In contrast, prostate histology was normal in doxycycline treated single transgenics and in untreated double transgenic mice (Fig. 1A).

**Figure 2:**
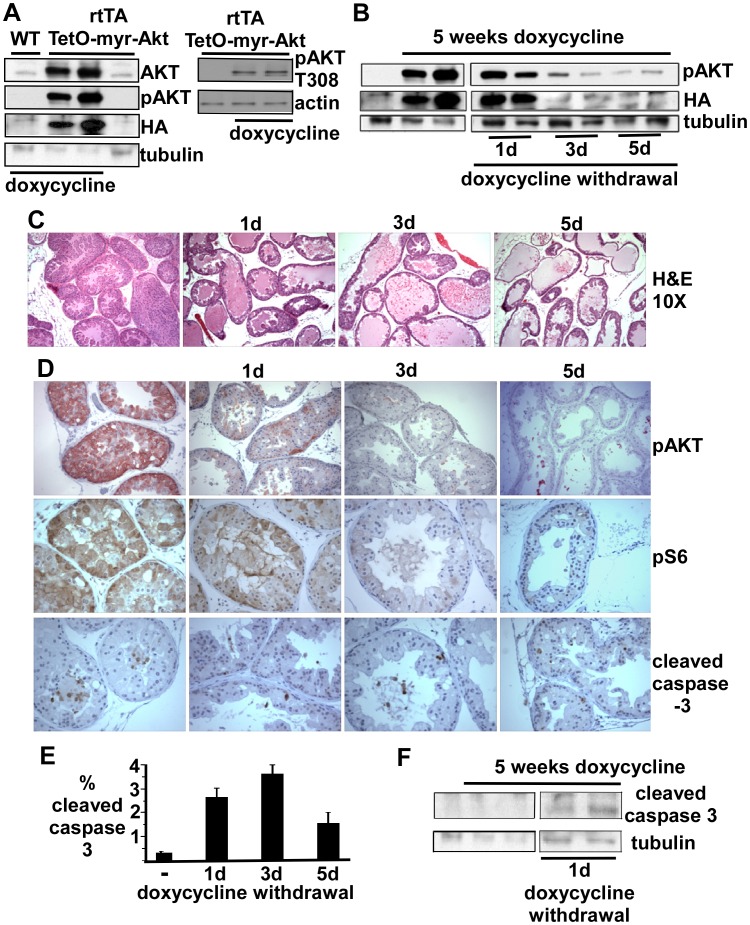
PIN lesions are rapidly reversed by doxycycline withdrawal. (**A,B**) Adult double transgenic mice or WT control mice were untreated or treated for 5 weeks with doxycycline and pairs of mice were then taken off of doxycycline for 0–5 days and sacrificed. Protein extracted from half of each ventral prostate was immunoblotted for pAKT (S473 or T308 where indicated), HA, and β-tubulin (loading control). (**C**) Histology of representative sections of ventral prostate after 0–5 days of doxycycline withdrawal. (**D**) Immunostaining of representative ventral prostate sections for pAKT (S473), pS6, and cleaved caspase-3. (**E**) Mean % cleaved caspase 3 positive epithelial cells counted in ten random fields from two mice at each time point. (**F**) Cleaved caspase 3 immunoblot of proteins extracted from ventral prostate in mice on doxycycline or 1 day after doxycycline withdrawal.

Immunostaining for the HA-epitope tag on myrAKT showed that the transgene was expressed specifically in regions showing hyperplasia (Fig. 1D). There was no detectable HA-staining in the absence of doxycycline treatment, and no detectable HA-AKT by immunoblotting (see Fig. 2). Moreover, immunostaining with an AKT pS473 antibody confirmed that the myrAKT was activated specifically in these hyperplastic/dysplastic regions. Analysis of additional animals given doxycycline for 3–5 weeks confirmed that hyperplasia was induced rapidly (data not shown). As reported previously in mice with constitutive ARR2Pb driven prostate epithelial expression of myrAKT [5], we did not observe progression of these PIN lesions to invasive cancer after doxycycline treatment for up to 8 months. Although senescence has been identified as a mechanism preventing progression in PI3 kinase/AKT pathway driven PCa mouse models, we have not yet seen clear increases in SA-β-galactosidase or HP1 in these PIN lesions (data not shown). Taken together, these results established that the ARR2Pb-rtTA transgene could stimulate doxycycline dependent expression of a tetO-regulated oncogene in prostate epithelium at functionally significant levels.

### Rapid Induction of Apoptosis by Doxycycline Withdrawal and myrAKT Downregulation

A previous study showed that PIN lesions in mice constitutively expressing myrAKT in prostate epithelium could be rapidly reversed by blocking mTOR complex 1 (TORC1) activity through treatment with a rapamycin analogue [14]. As this treatment does not selectively block transgene stimulated AKT pathway signaling and would abrogate basal TORC1 activity, we next determined whether removing doxycycline had acute effects on established PIN lesions. A series of double transgenic mice were treated with doxycycline for 5 weeks and then switched to water without doxycycline for 0–5 days. Immunoblotting for total AKT, pAKT and HA confirmed the doxycycline dependent high-level expression of the AKT transgene, and showed that it was phosphorylated at both S473 and T308 (Fig. 2A). Expression of the transgene started to decline by 1 day off doxycycline, and was markedly decreased by 3–5 days (Fig. 2B). Significantly, there was an associated rapid histological resolution of hyperplastic lesions, with the lumens initially filling with cellular debris and clearing by day 5 (Fig. 2C). The rapid decline in AKT activity was confirmed by immunostaining for pAKT, and by the rapid decline in phosphorylation of a downstream target, ribosomal protein S6 (Fig. 2D).

Interestingly, staining for cleaved caspase 3 prior to doxycycline withdrawal indicated that cells undergoing apoptosis were located primarily in the center of the hyperplastic glands (Fig. 2D). In contrast, apoptotic cells were readily detected throughout each gland after doxycycline withdrawal. These findings show that both suppression of apoptosis and increasing proliferation are critical functions of AKT in driving hyperplasia, although it may be contributing to hyperplasia by other mechanisms including suppression of autophagy or decreasing senescence. The fraction of cells staining with anti-cleaved caspase 3 is shown in figure 2E, and cleaved caspase 3 protein also could be detected by immunoblotting after doxycycline withdrawal (Fig. 2F). The apoptosis of cells in the center of hyperplastic glands prior to doxycycline withdrawal, in conjunction with ongoing proliferation (see Fig. 1C), suggest that apoptosis that is occurring despite high levels of AKT, in addition to cellular senescence, may be preventing further progression of these lesions.

### AR Protein Expression *in vivo* is not Downregulated by Induction of myrAKT

**Figure 3:**
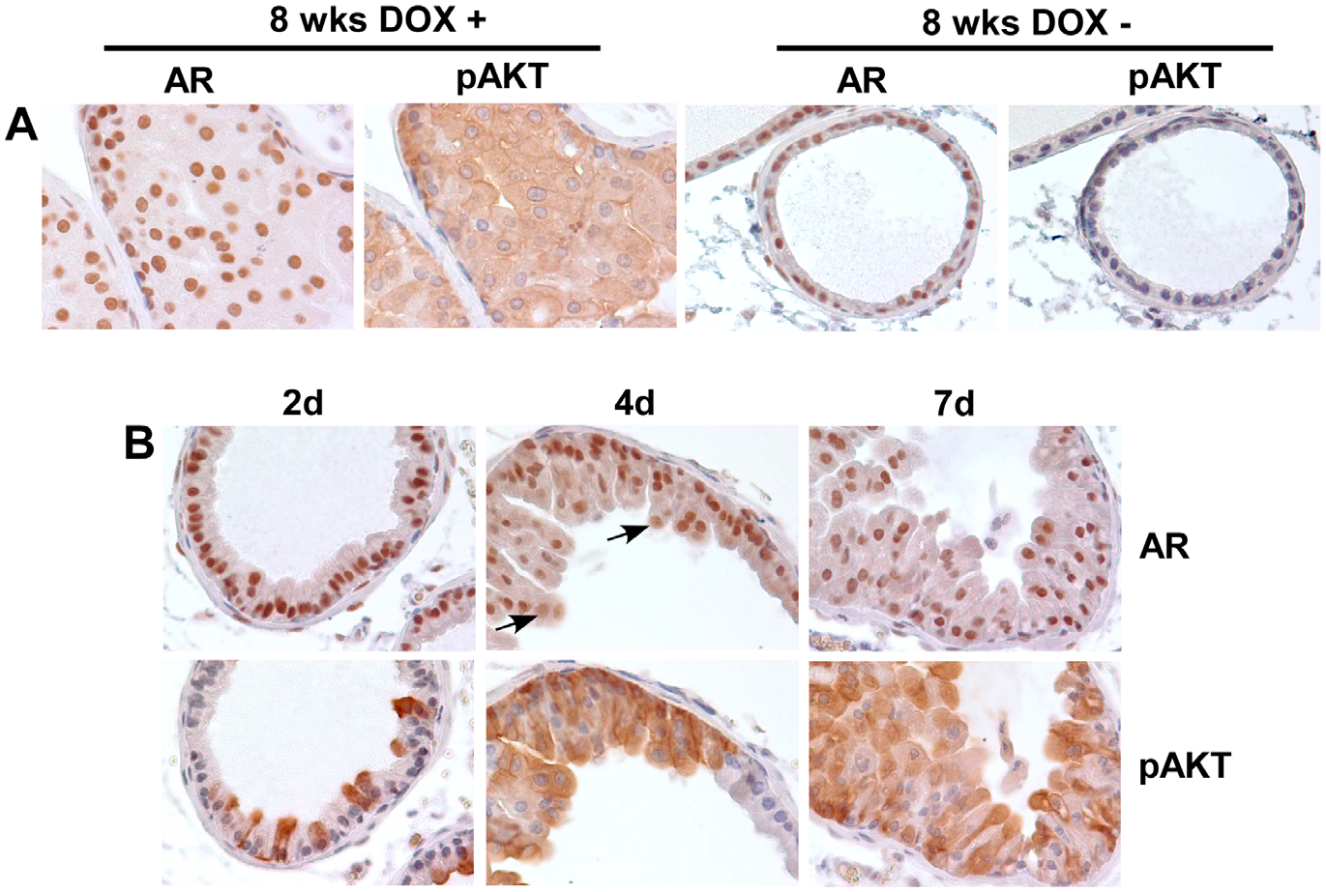
AR expression is not directly modulated *in vivo* in response to doxycycline induction of activated AKT. (**A**) AR and pAKT expression in ventral prostate from double transgenic mice induced or uninduced for 8 weeks. (**B**) AR and pAKT staining in serial sections of ventral prostate from double transgenic mice treated with doxycycline for 2, 4, or 7 days. Sections shown are representative of ventral prostates from at least 3 mice at each time. Arrows indicate examples of cells with large nuclei and lower AR that are not in contact with basal layer.

Previous *in vitro* studies have shown that AKT can phosphorylate serine 213 in the AR N-terminal domain, with subsequent ubiquitylation by MDM2 and proteasome mediated degradation [15], [16], [17], [18], [19]. However, the effects of AKT on AR expression appear to be variable and cell line dependent, and whether AR is a physiological target of AKT *in vivo* has not been determined. Therefore, we first examined AR expression by immunohistochemistry in PIN lesions from doxycycline treated double transgenic mice versus control mice, and found comparable high level nuclear expression of AR (Fig. 3A).

To determine whether AR may be downregulated acutely by activated AKT, we next examined AR expression in prostates after 2–7 days of doxycycline mediated myrAKT induction. Serial sections after 2 days on doxycycline showed induction of AKT in scattered luminal epithelial cells, without clear changes in AR expression (Fig. 3B). A larger fraction of cells expressed activated AKT after induction for 4 days, but AR expression was again not clearly decreased in the nuclei of these luminal epithelial cells located adjacent to the basal cell layer (although modest changes in AR levels may not be detectable by immunohistochemistry). A similar pattern was seen after 7 days of doxycycline induction. Interestingly, AR staining was less intense in many cells with larger nuclei that were starting to form an additional layer and were not in direct contact with the basal cell layer (examples indicated by arrows). These findings indicate that activated AKT does not directly cause marked changes in AR levels *in vivo*, but may indirectly alter AR expression through effects on proliferation or other pathways.

### AKT Induced Proliferation is Independent of p27 Cyclin Dependent Kinase Inhibitor Downregulation

A major mechanism through which AKT drives proliferation is phosphorylation and subsequent degradation of the p27 cyclin dependent kinase inhibitor. Significantly, a recent study in mice constitutively expressing an ARR2Pb regulated myrAKT transgene found that p27 was highly expressed in PIN lesions and contributing to senescence, and that crossing onto a p27 deficient background resulted in progression to invasive cancer [6]. Studies in conditional PTEN deficient mice have shown that progression from PIN to cancer is similarly suppressed by senescence, although through a p53 dependent pathway [1], [2], [3]. Nonetheless, p27 is highly expressed in these PTEN deficient senescent cells, and crossing onto a p27 deficient background also accelerates their progression to cancer [8]. These observations have indicated that PI3 kinase/AKT pathway activation may initially drive proliferation through mechanisms including p27 downregulation, but that additional mechanisms may subsequently increase p27 expression and result in cellular senescence.

**Figure 4:**
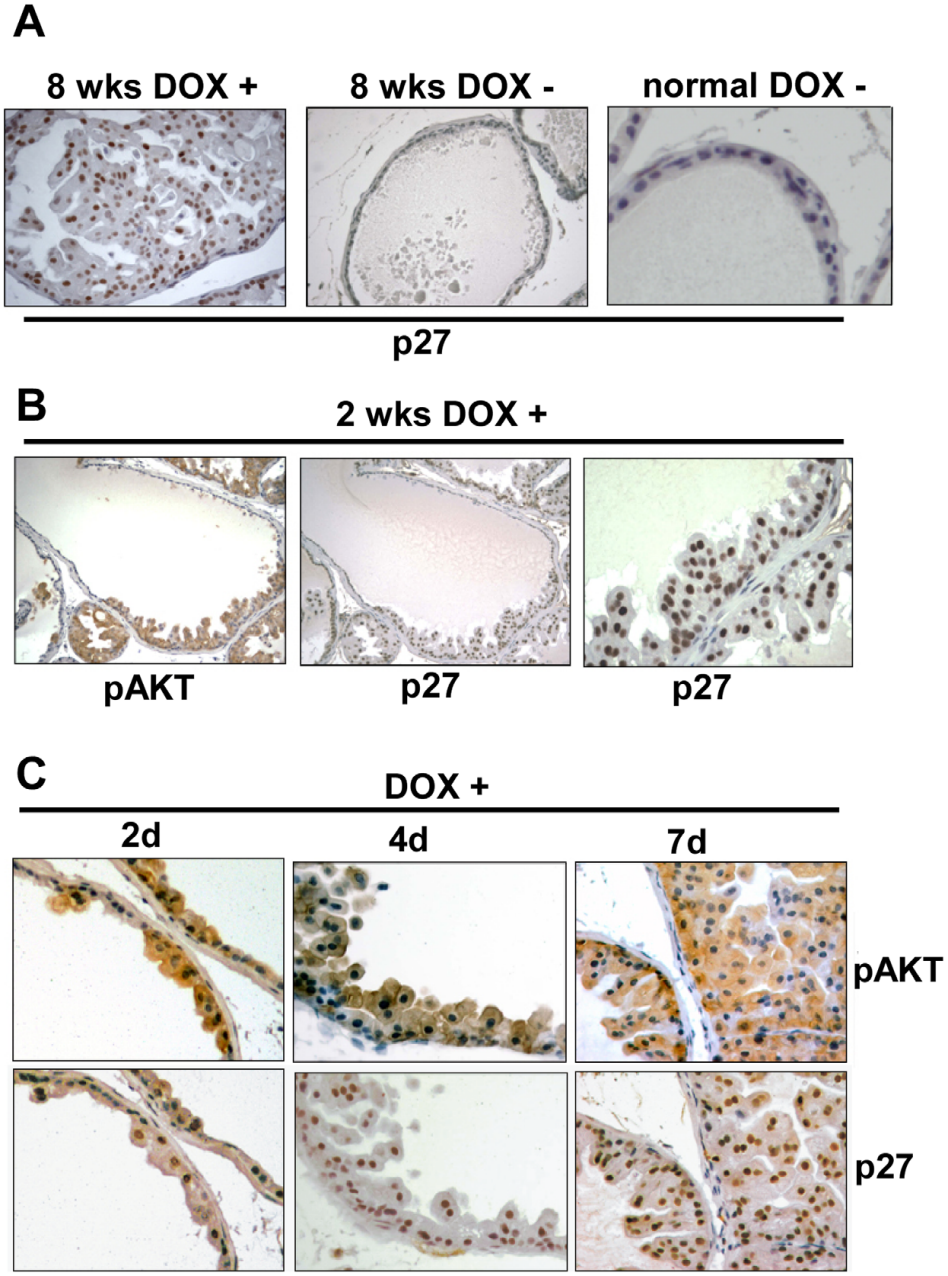
p27 expression is rapidly induced by doxycycline prior to development of PIN. (**A**) p27 immunostaining in ventral prostate of double transgenic mice treated for 8 weeks with or without doxycycline, and in prostate from a nontrangenic normal control (higher power). (**B**) p27 and pAKT expression in double transgenic ventral prostate after 2 weeks of doxycycline. (**C**) pAKT and p27 expression in ventral prostate of adult double transgenic mice treated with doxycycline for 2, 4, or 7 days. Sections shown are representative of ventral prostates from at least 3 mice at each time.

Consistent with these previous studies, we found that p27 was highly expressed in PIN lesions from mice treated with doxycycline for ∼8 weeks as compared to untreated or normal nontransgenic mice (Fig. 4A). However, increased p27 was also observed after doxycycline induction for only 2 weeks, and it was increased in regions expressing activated AKT without marked hyperplasia (Fig. 4B). Therefore, to more directly address whether AKT was initially downregulating p27, and whether a subsequent increase in p27 may be inhibiting progression of PIN lesions, we examined induction of myrAKT and p27 expression at very early times after treatment with doxycycline. As shown in figure 4C, activated AKT could be detected within 2 days of doxycycline treatment, while glandular hyperplasia was evident by 4 days. Significantly, p27 expression was not decreased in response to doxycycline, and was instead rapidly increased relative to adjacent glands that were not expressing the myrAKT transgene (Fig. 4C). Increased p27 was observed within 2 days in areas of AKT induction prior to hyperplasia, and at 4 days was observed in cells that were piling up into the lumen as well as in cells in contact with the basement membrane. There were no further clear increases in p27 expression at later time points or in PIN lesions, although the immunohistochemical staining is not quantitative and does not exclude further modest increases in p27 in PIN lesions.

### AKT Mediated p27 Downregulation in Murine and Human Cells *in vitro* is Dependent on T157

**Figure 5:**
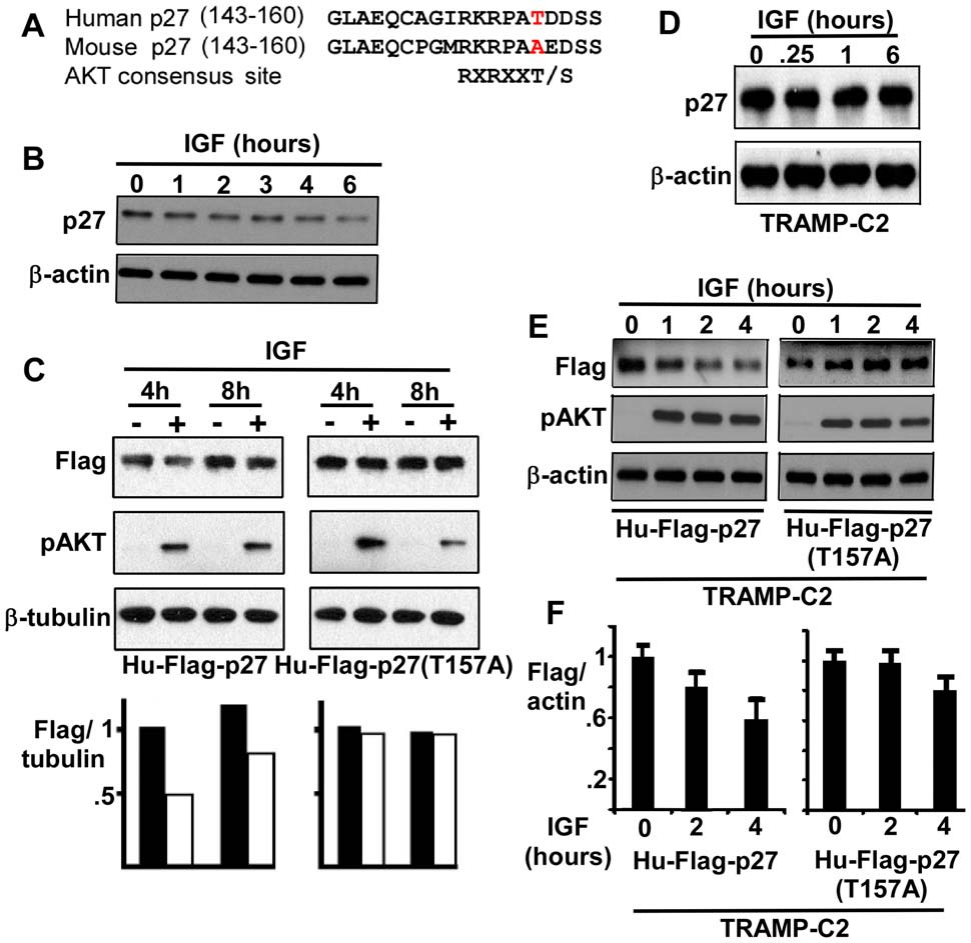
p27 downregulation in response to AKT activation is dependent on the nonconserved T157. (**A**) Sequences of human and murine p27. (**B**) Endogenous p27 expression in serum starved human CWR22Rv1 cells stimulated with IGF-1 for 0–6 hours. (**C**) Flag epitope tagged wild-type or T157A mutant human p27 expression vectors were transiently transfected into CWR22Rv1 PCa cells for 24 hours followed by overnight serum starvation. Cells were then treated with IGF-1 (10 ng/ml) as indicated, harvested after 4 or 8 hours, and lysates were immunoblotted for Flag-p27, pAKT, and β-tubulin (loading control). (**D**) TRAMP-C2 murine PCa cells were serum starved overnight and then IGF-1 stimulated as indicated. (**E**) TRAMP-C2 cells were transfected with Flag tagged human p27 expression vectors, serum starved overnight, and then treated with IGF-1 as in (**C**), followed by immunoblotting for Flag and pAKT. (F) Average Flag/actin ratios at 2 and 4 hours after IGF-1 stimulation (normalized to time 0) from three experiments were quantified.

Previous studies in human cells, including PCa cells, have shown that AKT enhances p27 degradation through phosphorylation of T157, which prevents nuclear translocation and thereby enhances degradation in the cytoplasm [20], [21], [22]. Significantly this site is not conserved in murine p27 (Fig. 5A), although AKT has been reported to target a distinct site in murine p27 [23]. To determine whether loss of this T157 site abrogates p27 downregulation in response to AKT activation in PCa cells, we transfected human CWR22Rv1 PCa cells [24] (expressing wild-type PTEN) with a human Flag-tagged wild-type or T157A mutant p27. Cells were then stimulated with IGF-1 to induce PI3 kinase/AKT activation and assessed for p27. As expected, IGF-1 stimulation decreased levels of the endogenous p27 in nontransfected CWR22Rv1 cells (Fig. 5B). IGF-1 stimulation similarly decreased levels of the transfected human wild-type p27 (Fig. 5C, representative of three experiments). In contrast, the T157A mutant was not decreased, supporting the conclusion that the absence of this site in mouse abrogates negative regulation by activated AKT.

We next examined a murine PCa cell line, TRAMP-C2, which was derived from the transgenic SV40 driven TRAMP model [25]. Consistent with the results in mouse prostate, PI3 kinase/Akt pathway stimulation by IGF-1 did not decrease p27 protein levels in these cells (Fig. 5D). To determine whether this was due to differences in murine versus human p27, we transiently transfected the TRAMP-C2 cells with the wild-type or T157A mutant human p27. Significantly, IGF-1 stimulation caused a decline in the wild-type, but not the mutant p27 (Fig. 5E, data from three experiments graphed in Fig. 5F). These results further support the conclusion that p27 is not an AKT substrate in murine prostate epithelium (Fig. 5D).

## Discussion

We initially confirmed that the ARR2Pb-rtTA mice could be used to rapidly and tightly control expression of the tetO-myrAKT oncogene. Induction of myrAKT in adult murine prostate epithelium resulted in PIN lesions that did not progress to invasive cancer, and the rapid induction of apoptosis and resolution of these lesions in response to doxycycline withdrawal confirmed that they remained dependent on the antiapoptotic functions of activated AKT. These results are consistent with previous data showing that PIN lesions in ARR2Pb-myrAKT mice respond rapidly to TORC1 inhibition by a rapamycin analogue [14]. Also consistent with previous observations, we observed high levels of p27 expression in established PIN lesions [5], [8]. However, examination of prostates at very early times after doxycycline induction revealed that p27 expression was already elevated in histologically normal myrAKT expressing cells and in very early hyperplastic lesions. We did not observe further increases in p27 in PIN lesions, but cannot rule out further small increases in PIN lesions that may contribute to preventing progression to invasive PCa. Nonetheless, these findings show that AKT driven proliferation in murine prostate epithelium is mediated by mechanisms that are independent of AKT mediated p27 downregulation.

AKT has been shown to downregulate nuclear p27 by at least two mechanisms in human cells. One mechanism is by phosphorylation of a consensus AKT site (T157), which prevents p27 nuclear translocation [20], [21], [22] and presumably enhances degradation by the cytoplasmic KPC ubiquitin ligase [26] and by the Skp1/Cullin-1/Skp2 E3 ubiquitin ligase complex that recognizes p27 subsequent to phosphorylation on T187 by CDK2 [27]. A second mechanism is by AKT1 mediated phosphorylation of a site on SKP2 (S72), which decreases its degradation and increases its cytoplasmic localization [28], [29]. Remarkably, these AKT sites on p27 and SKP2, although conserved in many species including humans and rats, are not conserved in mice.

While it remains possible that AKT regulates murine p27 and SKP2 expression directly or indirectly through alternative mechanisms in some cells or tissues [29], our results indicate that such alternative mechanisms regulating p27 expression are not prominent in murine prostate epithelium. Nonetheless, the marked hyperplasia in response to doxycycline induction of AKT that occurs despite high levels of p27 indicate that AKT may be blocking the CDK2 inhibitory function of p27 by novel mechanisms that remain to be identified. Alternatively, AKT phosphorylation and downregulation of p21 [30] or high level induction of D cyclin translation secondary to TORC1 activation [31] may overcome the inhibition of CDK2 by p27.

Interestingly, a dependence on induction of cyclin D translation downstream of TORC1 for proliferation in response to AKT activation in murine prostate epithelium would be consistent with the dramatic responses to TORC1 inhibition in mouse models [14], compared to the modest responses seen in humans where AKT can drive proliferation through p27 downregulation. Similarly, the inability of AKT to directly target p27 in mouse prostate may make p53 stimulated expression of p21 a more potent mechanism for suppressing tumor progression in mouse, and is consistent with the dramatic acceleration of tumor formation when *Pten* is lost on a p53 deficient background [1], [2]. Conversely, the ability of AKT to directly target p27 in human prostate may make it a more potent oncogene that can drive proliferation despite high level p53 induction of p21, and may in part explain why p53 loss is relatively uncommon as an early event in human primary PCa. In any case, these studies demonstrate a major difference between PI3 kinase/AKT pathway driven proliferation in human and murine cells that may be relevant for the interpretation of murine cancer models targeting this pathway and for the development of therapeutics.

## Materials and Methods

### Ethics Statement

This study was carried out in strict accordance with the recommendations in the Guide for the Care and Use of Laboratory Animals of the National Institutes of Health. All animal studies were approved by the Beth Israel Deaconess Institutional Animal Care and Use Committee (protocol #107-2010).

### Transgenic Mice and Cell Lines

A *Sac*II-*Bam*HI fragment encoding the rtTA from pUHDrtTA2S-M2 [12] was subcloned between the *Sac*II-*Bam*HI sites of pIRES2-EGFP (Clontech). The *Xho*I-*Not*I fragment containing rtTA-IRES-EGFP was then blunted and cloned into *Bam*HI digested and blunted pARR2Pb [11] to give the Pb-rtTA-IRES-EGFP. We confirmed androgen and tetracycline dependent activity by cotransfection with a tetO-luciferase reporter into AR expressing PCa cells (data not shown). The Pb-rtTA-IRES-EGFP fragment was excised for pronuclear microinjection in FVB (BIDMC Transgenic Facility). Founder lines with detectable transgene expression by RT-PCR in prostate were initially crossed with a tetO-β-galactosidase reporter strain. Lines with detectable β-galactosidase staining then were bred with tetO-myrAKT1 transgenic mice (HA-tagged), which were on a C57Bl/6 background so that the resultant mice were on a mixed FVB and C57Bl/6 background [13]. Transgene expression was induced by doxycycline (1 mg/ml) in the drinking water. To assess proliferation, mice were injected intraperitoneally with BrdU (0.1 ml of 10 mg/ml solution in sterile PBS) at 4 hours before sacrifice. CWR22Rv1 and TRAMP-C2 cells were obtained from the ATCC (characterization available at ATCC.org) and each were used within 30 passages (<4 months) of thawing. The expression vector for p27 (human pcDNA3-Flag-p27 wild-type) was generously provided by Dr. Masaki Mastsumoto (Kyushu University, Japan). The pcDNA3-Flag-p27(T157A) plasmid was constructed from this using the QuikChange Site-Directed Mutagenesis Kit (Stratagene).

### Immunoblotting and Immunohistochemistry

Prostate sections were snap frozen for protein extraction or formalin fixed. Proteins were extracted in RIPA buffer (50 mM Tris pH 8.0 with 1% TritonX-100, 0.5% deoxycholate, 0.1% SDS) and immunoblotted with anti-pAKT pS473 or pT308 (Cell signaling 193H12 or C31E5E, respectively, at 1∶1000 dilutions), anti-HA (Covance, 1∶1000 ), or anti-β-tubulin (Chemicon, 1∶2000). For immunostaining, slides were depariffinized, rehydrated, heated in boiling 10 mmol/L citrate buffer (pH 6.2) for 30 minutes, and blocked using 5% goat serum. For IHC, primary antibodies anti-pAKT (pS473, Cell Signaling, 1∶200), anti-HA (Cell Signaling, 1∶200 ), anti-pS6 (S240/244, Cell Signaling, 1∶2000), anti-cleaved caspase 3 (Cell Signaling, 1∶200), anti-p27 (#610242, BD Transduction Laboratories), or anti-AR (1∶100, Upstate) in 1% BSA were incubated overnight at 4°C, followed by biotinylated secondary antibody and streptavidin-HRP (1∶400, Vector). Slides were developed with 3,3-diaminobenzidine (DAB) and counterstained with hematoxylin. For IGF-1 stimulation, cells in 24-well plates were transfected with pcDNA3-Flag-p27(T157A) or pcDNA3-Flag-p27(WT) using Lipofectamine 2000. IGF-1 (InVitrogen) was added to 10 ng/ml, and cells were lysed in RIPA buffer with protease inhibitors and 100 mM Na_3_VO_4_.
